# Household Hints and Recipes

**Published:** 1875-01

**Authors:** 


### Household Hints & Recipes.

POLISHNG STOVES.—Stove lustre, when mixed with turpentine and applied in the usual manner, is blacker. more glossy, and more durable than when mixed with any other liquid. The turpentine prevents rust, and when put on an old rusty stove will make it look as well as new.

---

TO RENDER CARMINE MORE PURE AND BRILIANT.—Put the carmine in a solution of ammonia, and place in the sunshine. When the ammonia has acquired an intense blood red-color, it is poured off, and alcohol and acetic acid are added to it. The carmine is precipitated in a state of greatly increased brilliancy, the pure carmine being separated from the alumina.

---

A REDDISH-BROWN PAINT FOR WOOD.—The wood is first washed with a solution of 1 lb. cupric sulphate (blue vitriol) in 1 gallon of water, and then with ½ lb. potassic ferrocyanide (prussiate of potash) dissolved in 1 gallon of water. The resulting brown cupric ferrocyanide withstands the weather, and is not attacked by insects. It may be covered, if desired, with a coat of linseed-oil varnish.

---

TO FASTEN HANDLES ETC.—The following mixture is recommended for this purpose in the *Scientific American:* Mix together one pound of rosin and eight ounces of sulphur, and keep it either in bars or reduced to powder; mix one part of this powder with half a part of iron filings, fine sand, or brick dust. and the cavity of the handle is to be filled with this mixture. heat the stem of the knife or fork and insert it hot, and when cold it will be found tight.

---

HARDENING SMALL TOOLS.—According to J. Scheuszleder, watch-makers and engravers harden their tools in sealing wax. The article is made white hot and thrust into sealing-wax, allowed to remain a moment, then withdrawn and thrust into another place; and this treatment is continued until the steel is cold, and will no more enter the wax. The hardness thus obtained is extreme, and is comparable to that of the diamond; in fact, steel hardened in this way may be used for boring or engraving steel hardened by other processes, the tool being previously moistened with oil of turpentine.

A New Cement.—In the *Repertoire de Pharmacie*, M. Lallemand gives directions for making a new cement, which may be used for stopping the outlets of melting furnaces, lining water tanks, fixing stone in iron, etc.

Filings of cast or wrought iron....2 parts.
Chalk..........................10 parts.

These are mixed as intimately as possible, in a thousand of sal ammoniac, and then applied. It hardens slowly, adheres to all hard bodies, and mosture does not hinder it from setting.

---

To Cement Marble.—1. Melt together eight parts of resin and one of wax; when melted, stir in four or five parts of plaster-of-Paris. The pieces to be joined should be made hot.

2. Procure a small piece of quick-lime fresh from a newly-burnt kiln, slake with the white of an egg, wash the fractured parts quite clean, and apply.

3. Soak plaster of Paris in a saturated solution of alum, bake in an oven, reduce to powder, mix with water, and apply; it sets like granite.

---

Cement for Fastening Metals on Glass.—The following cement, reccommended by Franke, for fastening prize-medals, electrotypes of badges of honor, etc.,upon the show-cases in the Vienna Exposition, instead of the tedious and defective method of boring the glass, with its liability to fracture, may be found generally useful in fastening metal on glass securely, and rapidly. To an intimate mixture of two parts of finely-powdered silver litharge, and one part dry white lead, add as much of a mixture of three parts boiled linseed oil and one of copal varnish as will form a doughy mass. It is only necessary to cover the lower face of the medal with this cement, press it upon the glass, and remove the excess of cement.

---

Improved Starch.—A beautiful finish can, it is said, be given to articles to be starched by taking one-forth of a pound of starch, and working it over and kneading it with a little water, then placing five or six pints of water in a pan and adding to this a very small quantity of powdered borax, a small piece of sugar, and a fragment of white wax about the size of a hazel-nut, and heating the whole sufficiently. This water is then to be added to the starch, stirring it continually, and mixing the two together until the whole is as thick as is convenient for application. If the articles are to be made quite stiff, the strength of the starch may be increased two or three fold.

---

How to Soften Hard Putty.—A correspondent of *The Garden* says:—After many trials, and with a variety of differently shaped tools, with various successes, I at last accomplished my end by the simple application of heat. My first experiment was with a soldering iron, when to my great delight, I found the putty become so sóft that the broken glass could be removed by the fingers and the putty be easily scraped away. All that is required is a block of iron about two and a half inches long, by one and a half inches square, flat on the bottom.

---

Nutritive Meat Jelly.—Dr. Anstie, in his lectures on diseases of the nervous system, says that when it is necessary to give condensed nutriment in the smallest possible bulk, "nothing equals this jelly :—"

"Lean beef fillet, 3 pounds; lean veal, 3 pounds lean mutton, 3 pounds; cut up small and put into a saucepan *with no water;* simmer (never boiling) by the side of the fire for eight hours; strain the liquid (from a small quantity of tasteless insoluable fibre that remains), and let it jellify into a soft mass. This is an immensely concentrated *meat*, minus very little but the water which has been driven off; a teaspoonful or two of it is a wonderful support, and can be taken every hour with ease."

---

Liquid Glues.—A correspondent of the *Scientific American* gives the following in relation to liquid glues :

" An excellent liquid glue is made by dissolving glue in nitric ether. The ether will only dissolve a certain amount of the glue, consequently the solution cannot be made too thick. The glue thus made is about the consistency of molasses, and is doubly as tenacious as that made with hot water. If a few bits of India-rubber, cut into scraps the size of a buck-shot, be added, and the solution be allowed to stand a few days, being stirred frequently, it will be all the better, and will resist dampness twice a well as glue made with water. The best liquid glue that I have any knowledge of is made as follows :—Take gum of shellac three parts ; caoutchouc (India-rubber) one part, by weight. Dissolve the caoutchouc and shellac, in separate vessels, in ether free from alcohol, applying a gentle heat. When thoroughly dissolved, mix the two solutions, and keep in a bottle tightly stoppered. This glue is called marine glue, and resists the action of water, both hot and cold, and most of the acids and alkalies. Pieces of wood, leather, or other substances, joined together by it, will part at any point than at the joint thus made. If the glue be thinned by the admixture of ether, and applied as a varnish to leather, along the seams where it is sewed together, it renders the joint or seam water-tight, and almost impossible to separate.

---

Rosewood Stain.—Take 1 gallon alcohol, 1 lb. red sanders, 1 lb. dragon's-blood, 1 lb. extract logwood, ½ lb. gum shellac. Put the mixture into a strain and it will be ready for use. Apply with a brush, giving one, two, or more coats, according to the depth of color desired. Then give one or more coats of varnish. This stain is suitable for use on cane, willow, or reed work, and produces a good imitation of rosewood.

---

Colored Inks.—The following recipes have been well tested and are commended by good authorities as preferable to the solutions of aniline dyes which are now so extensively used as colored inks:—

*Green.* Two parts acetate of copper, one part carbonate of potash, and eight parts water. Boil till half is evaporated, and filter.

*Blue.* Three parts Prussian blue, one part oxalic acid, and thirty parts of water. When dissolved, add one part of gum-arabic.

*Yellow.* One part fine orpiment, well rubbed up with four parts thick gum-water.

*Red.* With the aid of a gentle heat, dissolve four grains of carmine in one ounce of aqua ammonia, and add six grains of gum-arabic.

*Gold.* Rub gold leaf, such as is used by bookbinders, with honey, till it forms a uniform mixture. When the honey has been washed out with water, the gold powder will settle at the bottom, and must be mixed with gum-water in sufficient quantity.

*Silver.* Silver-leaf treated in precisely the same manner gives a silver ink. Both these inks may, when dry, be polished with ivory.

*Black.* Three ounces crushed gall-nuts, two ounces crystallized sulphate of iron, two ounces gum-arabic, and twenty-four ounces water.

*White.* Fine French zinc-white, or white-lead, rubbed up with gum-water to the proper consistency.

A Simple Plan of Ventilation.—The following simple method for ventilating ordinary sleeping and dwelling rooms is recommended by Mr. Hinton in his "Physiology for Practical Use;" "A piece of wood, three inches high and exactly as long as the breadth of the window, is to be prepared. Let the sash be now raised, the slip of wood placed on the sill, and the sash drawn closely upon it. If the slip has been well fitted, there will be no draft in consequence of this displacement of the sash at its lower part; but the top of the lower sash will overlap the bottom of the upper one, and between the two bars perpendicular currents of air, not felt as draft, will enter and leave the room."

---

New Method of Preserving Grapes.—A French vine-dresser preserves grapes through the entire winter, their freshness, beauty, and savor remaining unimpaired, even until the month of April. He for a long time kept secret the process, but has lately given it to the world. The grapes are left upon the vine as late as possible, care being had, however, to cut them before the first frost. The bunch, in cutting, is not detached from the stalk or cane, but the latter is cut so that the cluster has attached to it, after cutting, two or three knots and joints below the cluster and two above. The upper end is then covered with wax, to prevent the evaporation of the fluids contained within the pores of the wood. All grapes, not absolutely healthy, are carefully removed from the cluster, after which the lower end of the stock is thrust through the hole in a perforated cork, and down into a bottle filled with water. In the water is a little wood-charcoal, which prevents it from becoming impure. The cork is crowded into the mouth of the bottle as closely as possible, and then covered with sealing-wax around the stalk, so as to close the bottle, water and air tight. The bottles are then placed upon tables or shelves in a dry chamber, in which the temperature never falls below the freezing point. The bottles are supported in any convenient way, so as to prevent their being tipped over by the weight of the clusters, and are placed at such intervals that the bunches do not come in contact with each other. The bunches must be, from time to time, carefully examined, and such single grapes as show symptoms of spoiling must be removed.—*American Artisan.*

